# Fulminant H1N1 and severe acute respiratory syndrome coronavirus-2 infections with a 4-year interval without an identifiable underlying cause: a case report

**DOI:** 10.1186/s13256-021-03113-9

**Published:** 2021-10-08

**Authors:** Terese L. Katzenstein, Sofie E. Jørgensen, Jann Mortensen, Marie Helleberg, Anna Kalhauge, Trine H. Mogensen

**Affiliations:** 1 Department of Infectious Diseases, Copenhagen University Hospital, Rigshospitalet, Esther Moellers vej 6, 2100 Copenhagen, Denmark; 2 Department of Infectious Diseases, Aarhus University Hospital, Aarhus, Denmark; 3 Department of Clinical Physiology and Nuclear Medicine, Copenhagen University Hospital, Rigshospitalet, Copenhagen, Denmark; 4 Department of Radiology, Copenhagen University Hospital, Rigshospitalet, Copenhagen, Denmark; 5 Department of Infectious Diseases, Aarhus University Hospital, Aarhus, Denmark

**Keywords:** SARS-CoV-2, COVID-19, H1N1, ICU, Interferon (INF)

## Abstract

**Background:**

The clinical presentation of severe acute respiratory syndrome coronavirus-2 infection is highly variable from asymptomatic infection to fulminant disease. The reasons for the variation are only starting to unravel, with risk factors including age and certain comorbidities as well as genetic defects causing immunological perturbations in the interferon pathways.

**Case presentation:**

We report the case of an otherwise healthy Caucasian man, who at ages 60 and 64 years suffered from severe H1N1 influenza virus infection and severe acute respiratory syndrome coronavirus-2 infections, respectively. In both cases, there were acute kidney impairment and the need for intensive care unit admission as well as mechanical ventilation. Fortunately, after both infections there was full clinical recovery. The severity of the infections indicates an underlying impairment in the ability to control these kinds of infections. Challenge of patient peripheral blood mononuclear cells showed impaired type I and III antiviral interferon responses and reduced interferon-stimulated gene expression. However, despite investigation of patient samples by whole exome sequencing and enzyme-linked immunosorbent assay, no known disease-causing genetic variants related to interferon pathways were found, nor were interferon autoantibodies demonstrated. Thus, any underlying immunological cause of this unusual susceptibility to severe viral infections remains unresolved.

**Conclusion:**

The patient experienced very similar severe clinical pictures triggered by H1N1 and severe acute respiratory syndrome coronavirus-2 infections, indicating an underlying inability to contain these infections. We were unable to show that the patient had any of the currently known types of immune incompetence but identified genetic changes possibly contributing to the severe course of both infections. Further analyses to delineate contribution factors are needed.

## Introduction

The clinical course of coronavirus disease 2019 (COVID-19) is highly variable. The reasons behind this are only now starting to be elucidated. Several clinical risk factors for severe COVID-19 have been identified, including older age, male sex, and various comorbidities [1, 2]. Furthermore, it has recently been shown that genetic defects in the toll-like receptor (TLR)-3 and -7 dependent type I interferon (IFN) pathway as well as IFN autoantibodies are highly enriched among patients with life-threatening COVID-19 [3–5]. Here, we report an otherwise healthy man who, with an interval of 4 years, suffered first fulminant H1N1 and later severe acute respiratory syndrome coronavirus 2 (SARS-CoV-2) infections, with full recovery after both, indicating an underlying weakness in the defense against these types of viral infections. Severe COVID-19 among individuals with prior fulminant viral infection(s) has, to our knowledge, not previously been reported.

## Case description

**Fig. 1:**
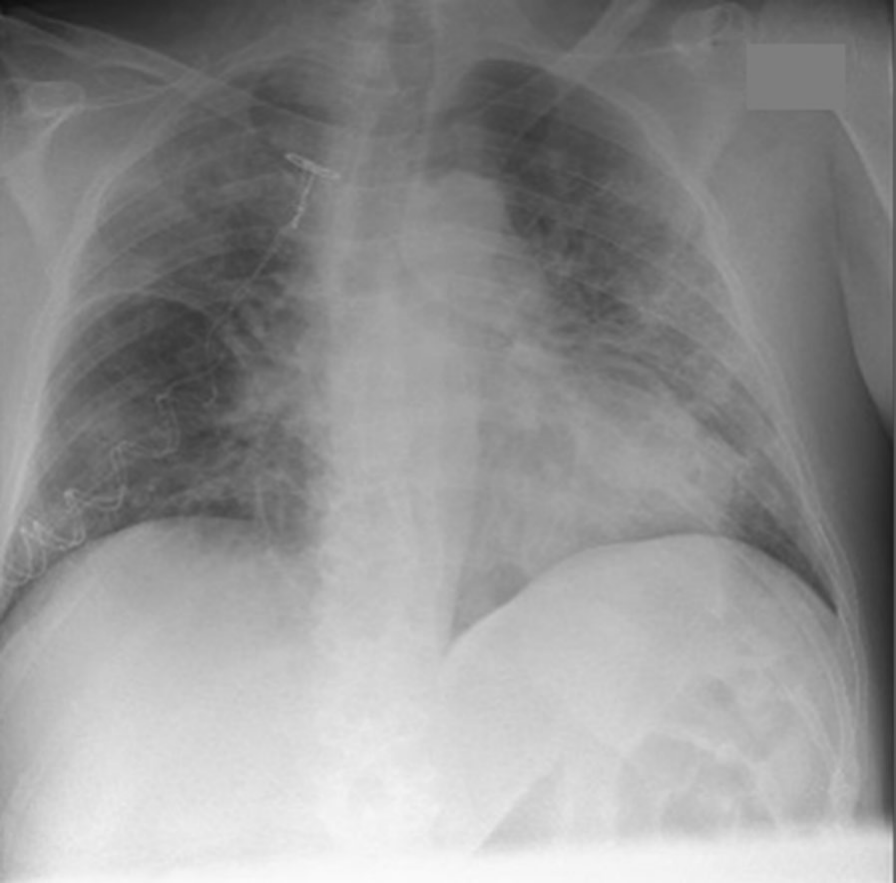
Chest X-ray obtained 1 day after readmission and diagnosis of SARS CoV-2 infection

A 64-year-old Caucasian man was admitted to a hospital in Copenhagen, Denmark with hematuria on 7 March 2020. Within one day of admission, he became febrile with respiratory symptoms interpreted as nosocomial pneumonia. Four days later, the patient was discharged with moxifloxacin scheduled for a follow-up transurethral prostatectomy. The patient was, however, readmitted with worsening respiratory symptoms on 13 March. Only then was he tested for SARS-CoV-2 by polymerase chain reaction (PCR) and tested positive. At admission, the patient had elevated inflammation markers: C-reactive protein of 231 mg/L (< 10 mg/L), slight neutrophilic leukocytosis of 8,7/10,3 × 10^9^/L (3.5–8.8 × 10^9^/L), and elevated lactate dehydrogenase of 526 U/L (105–205 U/L). Procalcitonin was not measured at admission but was elevated at 2.31 (< 50 μg/L) 4 days later. X-ray on 14 March showed bilateral infiltrates (Fig. 1). Due to respiratory deterioration, the patient was soon after transferred to the intensive care unit (ICU) with a sequential organ failure assessment (SOFA) score of 4 and an O_2_ requirement of 15 L. He rapidly deteriorated with hypoxic respiratory failure and needed mechanical ventilation (Drager V500, PS). One day later, extracorporeal membrane oxygenation (ECMO) was initiated and maintained for 6 days; during ECMO treatment the patient was heparinized. Subsequently, the patient was again placed on mechanical ventilation (highest *p* plateau 14 cm H_2_O, PS) for 3 days. After extubation, the O_2_ requirement steadily decreased, and no oxygen treatment was needed for the last 3 days of hospitalization. Neither steroids nor tocilizumab were given, as these treatments were not implemented as part of COVID-19 treatment at the time. Total duration of stay at the ICU and hospital was 12 and 24 days, respectively. During the COVID-19 infection, the patient developed kidney impairment with a maximum creatinine of 302 (reference 60–105) µmol/L. Within the following months, the patient recovered completely and was able to resume work full-time. Five months post-COVID-19, the lung diffusion capacity for carbon monoxide (*D*_LCO_) was mildly impaired (64% of predicted), while ventilation capacity was normal (forced expiratory volume in the first second 120% predicted, forced vital capacity 112% predicted, and total lung capacity 90% predicted) and respiratory muscle strength was normal (maximum expiratory and inspiratory pressure 72% and 100% predicted, respectively).

**Fig. 2:**
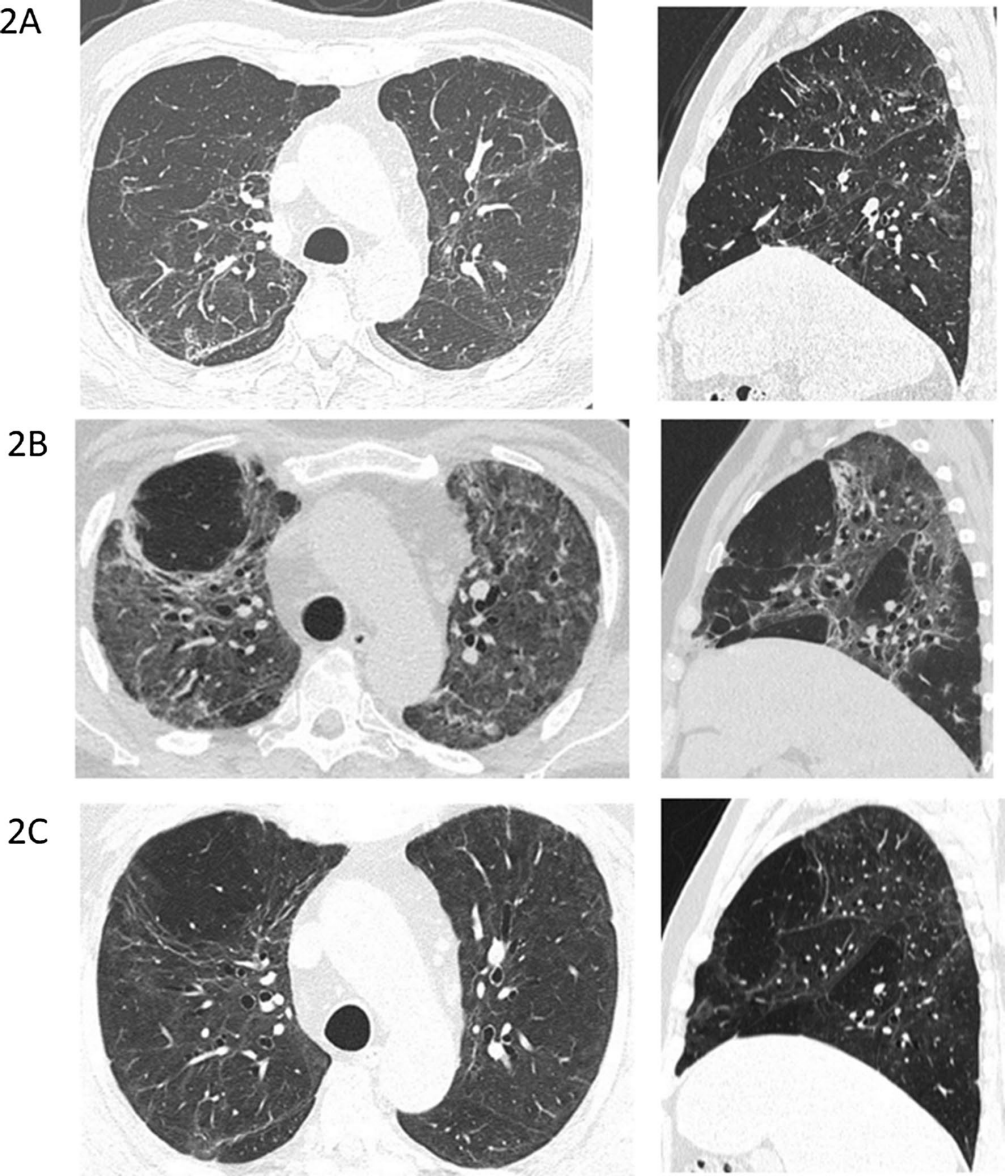
**A** CT scan at the end of the H1N1 infection with ground-glass opacities, reticulations, and traction bronchiectasis with upper region dominance. **B** CT scan 1 month after onset of COVID-19 infection showing a large extent of ground-glass opacities in both upper and lower regions, with a sparring of the subpleural regions. There is volume loss and bronchiectasis, all suggesting ongoing infection and inflammation with a component of organizing pneumonia. **C** CT scan 5 months after COVID-19 onset with almost normalization to the level of post-H1N1 infection

Interestingly, 4 years prior, the patient had experienced a very similar clinical and radiological picture (Table 2, Fig. 2a) due to an H1N1 infection requiring 5 weeks of hospital admission including ICU stay, mechanical ventilation, and dialysis. The patient had in the intervening period been without significant health problems except for known prostate hypertrophy and palpitations and marginally increased blood pressure treated with beta blockers. A CT scan had been performed at a follow-up post-H1N1 outpatient visit (Table 1). The findings included ground-glass opacities (GGO), reticulations, and traction bronchiectasis with an upper region dominance (Fig. 2A). No further scans were available prior to the patient being diagnosed with COVID-19. A CT scan 1 month after onset of COVID-19 revealed a large extent of GGO in both upper and lower regions. There was volume loss and bronchiectasis, all suggesting ongoing infection and inflammation with a component of organizing pneumonia (Fig. 2B). Five months after onset of COVID-19 symptoms, there was marked improvement, almost to the level of post-H1N1 infection (Fig. 2C).

**Table 1:** Timeline

Date of admission	Duration of admission, days	Diagnosis	SARS CoV-2 PCR positive	X-ray/CT scans
26 December 2015	36	H1N1		
27 March 2016		Follow-up post-H1N1		CT-scans (Fig. 2A)
7 March 2020	4	Hematuria, febrilia	Not done	
13 March 2020	24	COVID-19	–	CT-scans (Fig. 2B)
14 March 2020		COVID-19		X-ray (Fig. 1)
12 August 2020		Follow-up post-COVID-19		CT-scans (Fig. 2C)

**Fig. 3:**
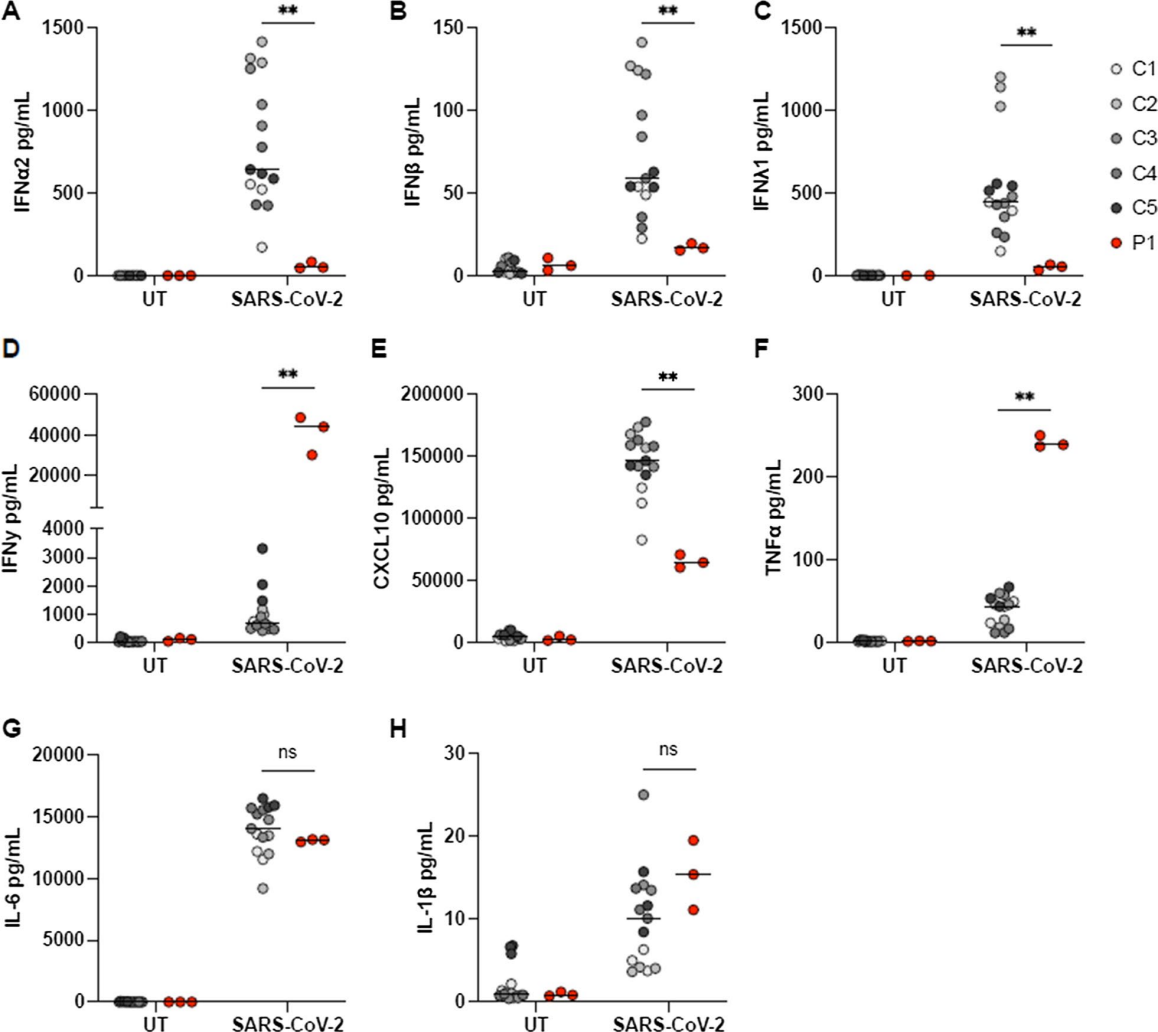
Cytokine responses in patient PBMCs upon SARS-CoV-2 infection. **A**–**H** Patient (P1) and control PBMCs (C1–5) were left untreated (UT) or infected with SARS-CoV-2 at a MOI of 0.5 for 24 hours. Induction of IFNs and proinflammatory cytokines was measured in supernatants by U-PLEX Meso Scale Technology. The experiment was performed once, but all stimulations were done in triplicate. Statistical differences were calculated using Mann–Whitney test. *p < 0.05, **p < 0.01, *ns* nonsignificant

Severe infection with H1N1 and SARS-CoV-2 have both been associated with genetic variants impairing the signaling pathways inducing type I IFN [3, 4]. We therefore investigated the patient’s ability to mount an innate immune response upon viral infection. PBMCs from P1 and healthy controls were infected with SARS-CoV-2 at a multiplicity of infection (MOI) of 0.5 for 24 hours, and the induction of IFNs and proinflammatory cytokines was measured in the supernatants. PBMCs from P1 demonstrated significantly impaired production of both type I and III IFNs (Fig. 3A–C), whereas the production of IFNγ was significantly increased compared with healthy controls (Fig. 3D). Patient PBMCs also exhibited significantly decreased CXCL10 levels in response to infection (Fig. 3E). Finally, of the three proinflammatory cytokines measured, only tumor necrosis factor alpha (TNFα) was significantly increased, as both interleukin 6 (IL-6) and IL-1β were expressed at levels comparable to the controls (Fig. 3F–H).

Despite the deficient type I and III IFN responses, genetic testing by whole exome sequencing for the recently reported disease-causing genetic mutations within the IFN signaling pathway [3, 4] was negative (see below). Additional rare gene variants with a high prediction for deleteriousness/disease-causing potential are presented in Table 2.

**Table 2:** Genetic variants

Gene	Transcript	Transcript variant	Protein variant	SIFT prediction	PolyPhen-2 prediction	CADD	MSC-CADD	GDI	GDI prediction	dbSNP ID	gnomAD frequency
STAT1	NM_007315.4	c.1371G>A	p.V457V	NA	NA	15.03	7.736	0.73972	Medium	144704615	0.028
PSMD2	NM_001278709.2	c.679G>A	p.G227S	Tolerated	Probably damaging	26	6.072	4.97534	Medium	NA	0.000
CFI	NM_000204.5	c.1019T>C	p.1340T	Damaging	Possibly damaging	22.3	0.001	2.21508	Medium	769419740	0.007
PCDH18	NM_019035.5	c.3154G>A	p.G1052R	Damaging	Possibly damaging	23.7	3.313	2.92063	Medium	148983038	0.002
SNX9	NM_016224.5	c.199C>G	p.Q67E	Tolerated	Benign	16.55	3.313	2.29269	Medium	749203434	0.002
DOCKS	NM_001190458.2	c.499C>G	p.Q167E	Tolerated	Benign	18.27	0.001	18.28926	High	1233535978	0.000
GAS6	NM_000820.4	c,451C>T	p.R151W	Damaging	Probably damaging	32	3.313	3.51157	Medium	202152067	0.004
FANCI	NM_018193.3	c.2017C>T	p.H673Y	Damaging	Probably damaging	25.2	0.001	17.44782	High	763866498	0.003
P2RX5	NM_175080.3	c.88G>T	p.G30C	Damaging	Probably damaging	28	3.313	2.65609	Medium	NA	0.000
SBNO2	NM_001100122.2	c.1195G>A	p.E399K	Tolerated	Probably damaging	25.2	3.313	5.7817	Medium	1432406684	0.000
SLC44A2	NM_001145056.2	c.2027A>T	p.N6761	Damaging	Probably damaging	26.9	3.313	2.4366	Medium	768714484	0.004

Genetic variants were identified in the patient using the following criteria: CADD score > 15 and GnomAD frequency < 0.1%. Overview of gene name, transcript variant, protein variant, SIFT function prediction, PolyPhen-2, variant ID, gnomAD frequency, CADD score, MSC, GDI score, GDI prediction, and inferred activity

*CADD* combined annotation dependent depletion, *MSC* mutation significance cut-off, *SIFT* sorting intolerant from tolerant, *MSC* mutation significance cut-off, *gnomAD* genome aggregation database, *GDI* gene damage index

Finally, presence of IFN autoantibodies has been demonstrated in plasma from up to 10% of cases with severe COVID-19 [4], thereby functionally impairing IFN activities, in the absence of disease-causing genetic variants. However, investigation for autoantibodies against IFNα or IFNω in patient plasma by ELISA did not reveal the presence of such autoantibodies (data not shown).

## Discussion

We report the case of a man who, at ages 60 and 64 years, suffered severe H1N1 and SARS-CoV-2 infections, respectively, in both cases with acute kidney injury and requirement for mechanical ventilation. Both prior to the H1N1 infection and pre- and post-COVID-19, the patient was without respiratory complaints and, if any, only marginal, renal impairment. Lung function test 5 months post-COVID-19 was with slightly reduced *D*_LCO_ at 64% of expected, while FEV1 and FVC were at 120% and 112% of predicted, respectively. Among the post-COVID-19 lung function test impairments most often reported is reduced *D*_LCO_ [6, 7], in line with the findings in the current case. The radiological findings were also in line with those reported in the literature [8, 9].

The reason why this otherwise healthy man was so severely affected by these viral infections is currently undetermined. Infection of patient PBMCs with SARS-CoV-2 *in vitro* demonstrated significantly impaired type I and III IFN as well as CXCL10 responses, which may have resulted in impaired ability to clear the infection. Unfortunately, we were not able to couple the impaired responses with genetic variants in any of the IFN pathway susceptibility genes previously reported, including IRF3, IRF7, IRF9, TLR3, TRIF, TRAF3, UNC93, TBK1, IFNAR1, STAT1, and STAT2 [3]. We did, however, identify variants in other genes that could potentially be of relevance (Table 2), although these were not functionally validated, therefore leaving a potential link to the disease presentation uncertain.

Most likely, further inborn errors of immunity and/or acquired traits will be identified as explanations for the highly variable clinical course among individuals infected with SARS CoV-2.

The contribution to COVID-19 case severity impacted by various immunodeficiencies is largely unresolved. It has been debated whether certain immunodeficiencies might paradoxically protect against severe disease [10]. A British study found an overall negative effect of immunodeficiencies, with secondary immunodeficiencies resulting in worse clinical courses relative to primary immunodeficiencies [11]. Other studies have also found certain immunodeficiencies predisposing to more severe disease [12]. Further unraveling of the interplay between the various types of immune incompetence and clinical course of COVID-19 will most likely further our understanding of both the pathogenesis of SARS CoV-2 and the functioning of the various components of our immune system.

It is reassuring that even patients suffering from full-blown infections (*in casu* to H1N1 and SARS CoV-2) can fully recover. However, the requirement for ICU treatment for these individuals, for which access is limited or lacking in many countries highly affected by the current COVID-19 pandemic [13, 14], is worrisome. Hopefully, potent vaccines [15, 16] and treatments will soon be widely available, minimizing the need for ICU treatment.

### Patient perspective

The patient reported satisfaction with the treatment received during admission for COVID-19 and during follow-up.

## Conclusion

We report a case where a patient experienced very similar severe clinical pictures triggered by H1N1 and SARS-CoV-2 infections, indicating an underlying inability to contain these infections. We were unable to show that the patient had any of the currently known types of immune incompetence but identified genetic changes possibly contributing to the severe course of both infections.

## Materials and methods

### PBMC purification

PBMCs were purified from heparinized blood by Ficoll density centrifugation. Isolated PBMCs were stored in liquid nitrogen until further use. PBMCs were cultured in Roswell Park Memorial Institute (RPMI) medium with 10% heat-inactivated fetal bovine serum and 1% penicillin–streptomycin.

### DNA isolation

DNA was isolated from ethylenediaminetetraacetic acid (EDTA)–blood using the QIAamp DNA Blood Mini Kit (Qiagen, 51104).

### Whole exome sequencing analysis

Whole exome sequencing was performed as previously described [17]. Variants were kept only if minor allele frequency < 0.1%, CADD > 15. Loci associated with severe COVID-19 described by Zhang *et al*. [3] were included in the variant filtering.

### SARS-CoV-2 infection

Patient and control PBMCs were seeded in 96-well plates (500,000 per well), rested overnight, and subsequently infected with SARS-CoV-2, strain FR-4286, at an MOI of 0.5 for 24 hours. Supernatants were harvested, inactivated with 0.4% Triton-X-100 at a ratio of 1:1 for 30 minutes, and stored at − 80 °C until further use.

### Meso Scale

Cytokine expression was measured in supernatants from SARS-CoV-2-infected PBMCs by U-PLEX Meso Scale assays detecting IFNα2a, IFNβ, IFNγ, IFNλ1, TNFα, IL-1β, L-6, and CXCL10 (K15067L-2, MSD) according to the manufacturer’s instructions.

### IFN autoantibodies

IFN autoantibodies were measured in plasma as previously described [4]. Briefly, ELISA plates were coated with 1 μg/mL IFNα (130-093-874, Miltenyi Biotec) or IFNω (BMS304, Invitrogen) overnight at 4 °C followed by blocking in 5% skimmed milk. Plasma samples were diluted 50× in high-performance ELISA (HPE) buffer (M1940, Sanquin) before incubation on the plates. Bound autoantibodies were detected with horseradish peroxidase (HRP)-conjugated goat anti-human IgG IgA IgM (GAHu/Ig(Fc/PO, Nordic-MUbio) and HRP substrate SureBlue KPL (5120-0077, SeraCare).

### Statistics

Experiments were performed once due to limited patient material, but all stimulations were done in triplicate. Statistics were calculated using Mann–Whitney test.

## Data Availability

Data available to researchers upon request.

## Abbreviations

COVID-19: Coronavirus disease 2019
CT scan: Computer tomography scan
D_LCO_: Lung diffusion capacity for carbon monoxide
ECMO: Extracorporeal membrane oxygenation
FEV1: Forced expiratory volume in the first second
FVC: Forced vital capacity
GGO: Ground-glass opacities
H1N1: Hemagglutinin 1, neuraminidase 1
ICU: Intensive care unit
INF: Interferon
PBMC: Peripheral blood mononuclear cells
SARS-CoV-2: Severe acute respiratory syndrome coronavirus 2
TLR: Toll-like receptor
